# Supplementary material for: Obligate endosymbiosis enables genome expansion during eukaryogenesis
Source: Commun Biol. 2023 Jul 25;6:777. doi: 10.1038/s42003-023-05153-x (PMC10368719; doi:10.1038/s42003-023-05153-x)
Supplement: Supplementary file 1 — Supplementary Information [file 42003_2023_5153_MOESM1_ESM.pdf]

Supplementary Material for Obligate Endosymbiosis Enables Genome Expansion During Eukaryogenesis

Samuel H. A. von der Dunk[1*], Paulien Hogeweg[1], Berend Snel[1]

[1]Theoretical Biology and Bioinformatics, Department of Biology, Science Faculty, Utrecht University

Padualaan 8, 3584 CH, Utrecht, The Netherlands

[*]Email: s.h.a.vonderdunk@uu.nl

July 6, 2023

Supplementary Note 1

Genome size evolution directs holobiont adaptation

Genome size evolution is not just the byproduct of selection for cell-cycle coordination. In several replicates, a marked switch in holobiont behavior evolves after extensive symmetry breaking in genome size. In two replicates, C11 and C12, the evolutionary trajectories intersect in phenotypic space (Fig. S1): C11 evolves from low cell density and low symbiont number (r-strategy) to high cell density and high symbiont number (K-strategy), and C12 follows this path in reverse. In both cases, competition experiments between populations at different times show that each evolutionary path is entirely adaptive from before until after the switch, and this is supported by the congruence between technical replicates.

The critical difference between C11 and C12 which explains why seemingly identical holobionts (around $t = 0.5 \cdot 10^6$) continue to evolve in diametrically opposite directions, is the distinct cell-cycle behavior that underlies growth coordination. In C11, holobionts are formed by a generalist host (R8) and a specialist symbiont (R3) resembling P9, whereas in C12, holobionts are made up of a specialist host (R3) and a generalist symbiont (R8; as clearly seen in the top and bottom right-most panels in Fig. S1). These contrasting host–symbiont behaviors can generate very similar growth dynamics (i.e. phase diagram at $t = 0.5 \cdot 10^6$), but ultimately give rise to different constraints and different opportunities for adaptation. Genome size asymmetry is the key factor that opens up the different adaptive

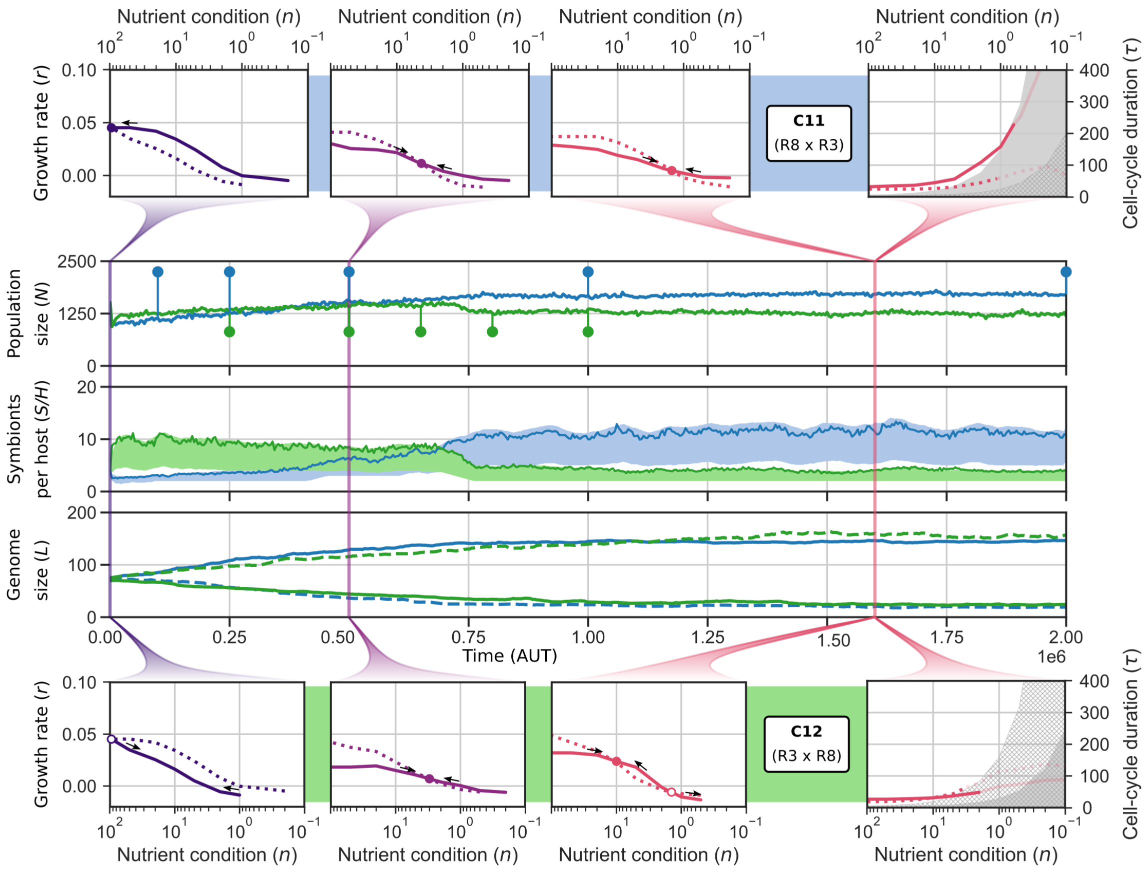

Figure S1: Strategy switching through symmetry breaking in genome size in C11 and C12. These two populations follow opposite trajectories, and intersect in several aspects: population size, symbiont number and growth dynamics. For each replicate, competition experiments between populations from different timepoints (circles in population size panel) revealed unequivocal fitness increase across the strategy switch. The cell-cycle behavior (top and bottom right-most panels) show that cell-cycle behavior is very different between C11 and C12, explaining why holobionts traverse opposite evolutionary trajectories. For symbionts per host (middle panel), the shaded area spans the 33% and 67% quantiles; the lines show the means (distributions are skewed).

routes: when we disallow sharing of household genes between host and symbiont, genome size asymmetry is less extreme and the strategy switches do not occur.

On the long term, the trajectories of C11 and C12 are not equally adaptive. The final population of C11 (at $t = 10^7$) outcompetes that of C12, reiterating that holobionts with large host and small symbiont genomes—i.e. those resembling eukaryotes—are fitter. More specifically, the r-strategy yields small symbiont populations with very large genomes, which weakens selection and reduces competitiveness late in the experiment (around $t = 8 \cdot 10^6$).

Supplementary Tables and Figures

Table S1: Important characteristics of pre-evolved free-living prokaryotes, i.e. cell-cycle efficiency (see Fig. 6) and generalist capacity (plasticity measured as the log-difference between cell-cycle duration at $n = 0.1$ and $n = 100$).

Strain	Efficiency (e)	Plasticity (σ)
R8	0.476	0.965
R9	0.402	0.866
R2	0.307	-0.355
R3	0.160	0.497

Table S2: Host–symbiont pairs used to initialise evolution experiments (see Table S1 for characteristics of prokaryotes). C1–4 start with identical host and symbiont; C5–6 have different host and symbiont but with very similar phenotypic behavior, i.e. R8 and R9 are both efficient generalists; C7–12 are asymmetric.

Holobiont	Host	Symbiont
C1	R8	R8
C2	R9	R9
C3	R2	R2
C4	R3	R3
C5	R8	R9
C6	R9	R8
C7	R8	R2
C8	R2	R8
C9	R3	R2
C10	R2	R3
C11	R8	R3
C12	R3	R8

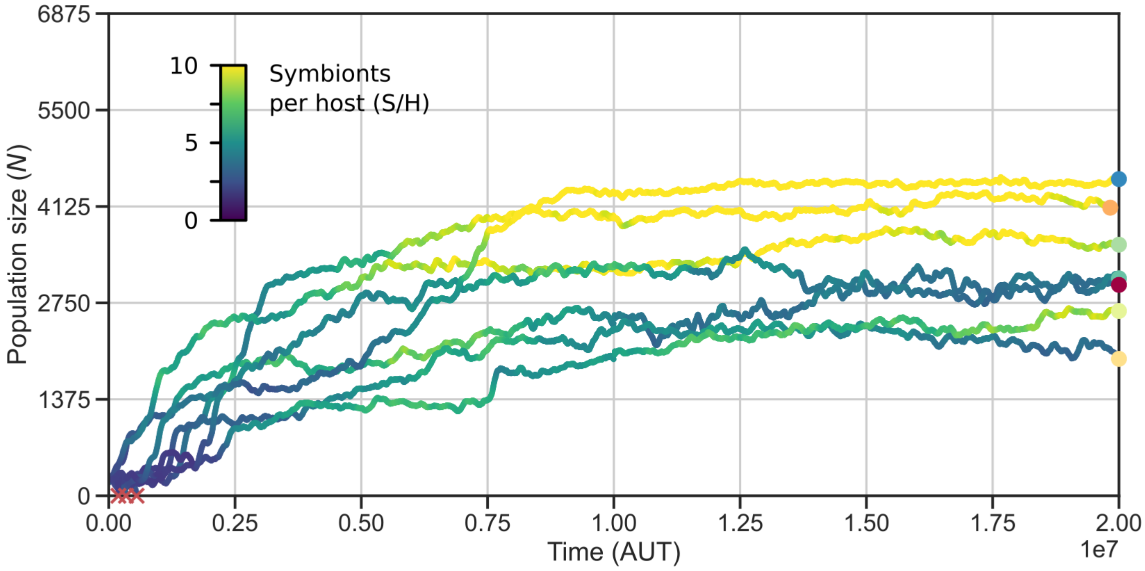

Figure S2: Evolutionary trajectories of the replicates with primitive FECA, continued until $t = 2 \cdot 10^7$, showing that adaptation saturates after $t = 10^7$ (cf. Fig. 2a).

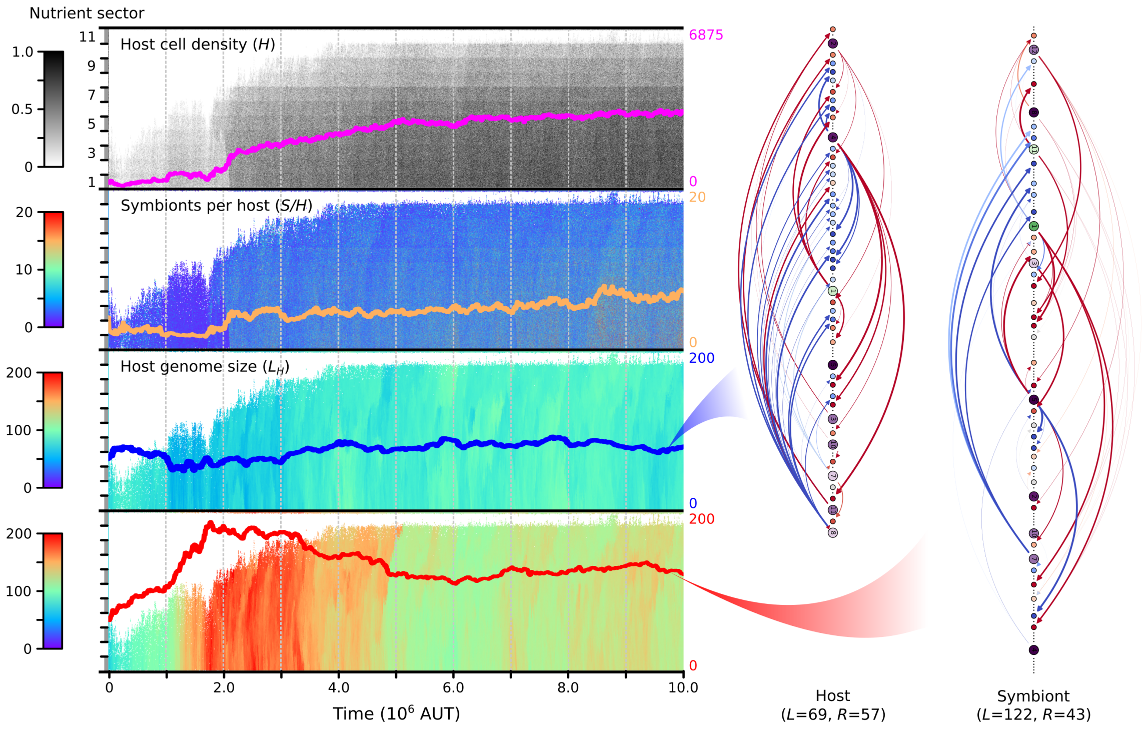

Figure S3: Evolutionary trajectory of P8, i.e. an alternative outcome of evolution from primitive FECA (cf. Fig. 3). Here, holobionts evolve an r-strategy (moderate symbiont numbers) and large symbiont and small host genomes. Symbiont genomes expand dramatically early on when holobionts are still barely viable. The first major adaptation happens at $t \approx 2 \cdot 10^6$, coinciding with an increase in symbiont numbers. Afterwards, symbiont numbers and population size increase only slightly and never reach the levels that characterize the K-strategy as seen in P9. Interestingly, the regulatory repertoire of the host remains larger than that of the symbiont, suggesting that more complex regulation is generally required for the host than for the symbiont, even when a small host genome size is favored.

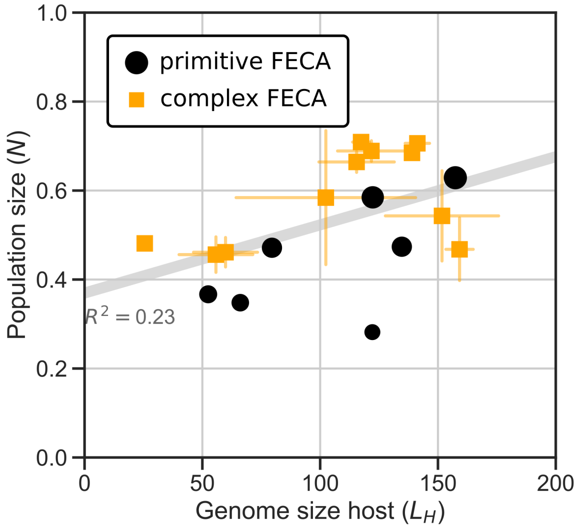

Figure S4: Holobionts with larger host genomes, i.e. eukaryote-like holobionts, have adapted more successfully and achieved greater population size ($p = 0.046$, $N = 18$). For the experiment initialised with complex FECA, 2-3 technical replicates have been averaged and error bars show standard deviations in both dimensions. For the experiment initialised with primitive FECA, the size of the markers is scaled by final population size.

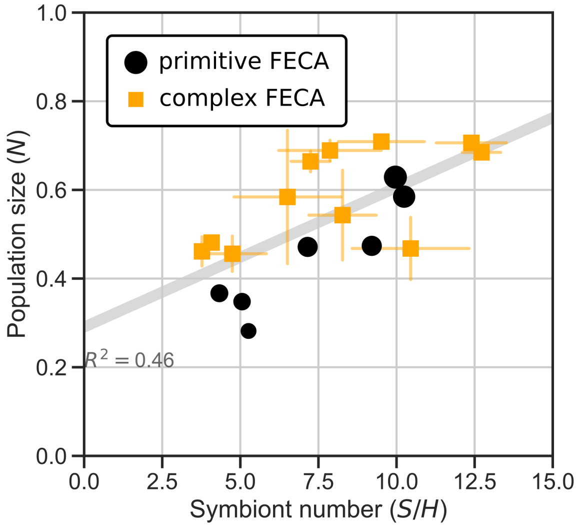

Figure S5: Final population size ($t = 10^7$) correlates with symbiont number across evolution replicates all replicates ($p = 0.0019$, $N = 18$). For the experiment initialised with complex FECA, 2-3 technical replicates have been averaged and error bars show standard deviations in both dimensions. For the experiment initialised with primitive FECA, the size of the markers is scaled by final population size.

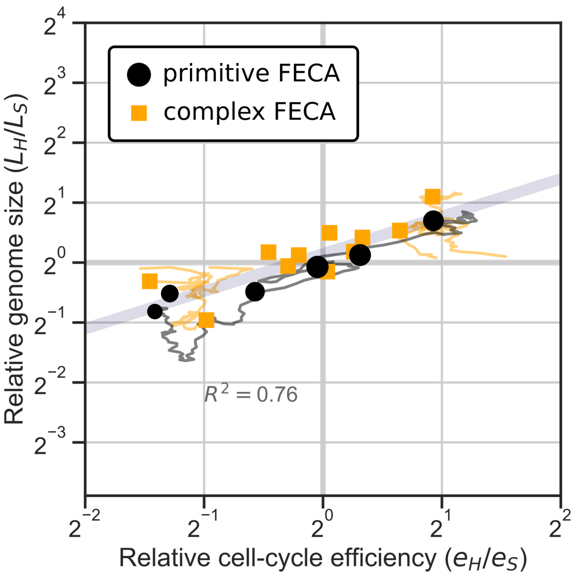

Figure S6: Asymmetry in cell-cycle efficiency yields genome size asymmetry, even when household genes cannot be shared between host and symbiont ($p = 4.43 \cdot 10^{-6}$, $N = 17$). In the presence of non-adaptive forces alone, genome size asymmetry is much less extreme (cf. Fig. 6). The markers show the final timepoint of the experiment. Here, only one technical replicate is shown for each of the replicates initialised with complex FECA. For the experiment initialised with primitive FECA, the size of the markers is scaled by final population size.
